# Multi-Object Tracking with Correlation Filter for Autonomous Vehicle

**DOI:** 10.3390/s18072004

**Published:** 2018-06-22

**Authors:** Dawei Zhao, Hao Fu, Liang Xiao, Tao Wu, Bin Dai

**Affiliations:** 1 College of Artificial Intelligence, National University of Defense Technology, Changsha 410073, China; zhaodawei12@nudt.edu.cn (D.Z.); fuhao@nudt.edu.cn (H.F.); wutao@nudt.edu.cn (T.W.); 2 National Innovation Institute of Defense Technology, Beijing 100091, China; xiaoliang@nudt.edu.cn

**Keywords:** multi-object tracking, correlation filter, convolutional neural network, autonomous vehicle

## Abstract

Multi-object tracking is a crucial problem for autonomous vehicle. Most state-of-the-art approaches adopt the tracking-by-detection strategy, which is a two-step procedure consisting of the detection module and the tracking module. In this paper, we improve both steps. We improve the detection module by incorporating the temporal information, which is beneficial for detecting small objects. For the tracking module, we propose a novel compressed deep Convolutional Neural Network (CNN) feature based Correlation Filter tracker. By carefully integrating these two modules, the proposed multi-object tracking approach has the ability of re-identification (ReID) once the tracked object gets lost. Extensive experiments were performed on the KITTI and MOT2015 tracking benchmarks. Results indicate that our approach outperforms most state-of-the-art tracking approaches.

## 1. Introduction

Multi-object tracking is one of the most fundamental capabilities for the autonomous vehicle. The autonomous vehicle mostly works in the dynamic environment. Only through precisely tracking other dynamic objects’ movements, the autonomous vehicle could plan its own trajectory and run smoothly.

Existing multi-object tracking approaches mostly adopt the tracking-by-detection strategy. This is a two-step procedure. In the first step, the potential objects-of-interest are detected using object detection algorithm. These potential objects are then linked across different frames to form the so-called tracklets in the second step.

Recently, one has witnessed great improvements in object detection field. One of the most influential works is the region proposal based Convolutional Neural Network (CNN) [1,2,3,4]. This approach firstly generates object proposals using Region Proposal Network (RPN), and then features are extracted for each proposal. To detect multi-scale objects, the proposals are usually generated from the deep layers, such as the conv-5 layer [3]. However, the spatial resolution of the conv-5 layer is only 1/16 of the original image, making the algorithm difficult to generate small object proposals [5]. To tackle this problem, Liu et al. [6] proposed a Single-Shot multibox Detector (SSD) which gets rid of the region proposal branch. This approach directly generates object proposals on multiple layers. Small object proposals can be generated on shallow layers, and large objects could be generated on deep layers. Nonetheless, as shallow layers only contain low-level features, the small object proposals usually contain many false detections.

**Figure 1:**
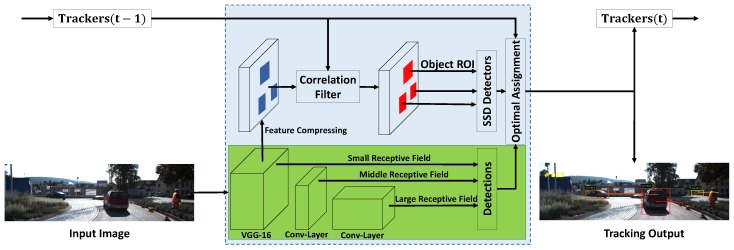
The framework of the proposed approach.

In the context of multi-object tracking, the problem of detecting small objects might be alleviated by utilizing temporal information. In this paper, our first contribution is to improve the single-frame object detection algorithm by incorporating temporal information. More specifically, we propose a multi-scale object detector that augments the SSD with temporal Region of Interests (ROIs), as shown in Figure 1. The proposed algorithm generates object proposals in the ROIs predicted by the tracker. The ROIs are then resized into a fixed size. In this way, small objects can be upscaled larger and the corresponding proposals can be generated on upper layers. Therefore, for the small object, SSD can exploit the deep discriminative features, which is proven to be beneficial for reducing the false detections.

Once the objects are detected, they are then linked across different frames using data association algorithm [7,8,9]. No matter what the data association algorithm is, it always relies on an affinity model which describes the similarity of detections. Many recent works try to build robust affinity models.

In [10], the authors proposed a new descriptor named as Aggregated Local Flow Descriptor (ALFD). This descriptor is then used with the motion relation model to perform as the affinity model. The motion relation model describes motion relations between bounding boxes. However, this model only works for a static camera. In [11], the authors used Integral Channel Features (ICF) to incrementally train a Multiple-Instance Learning (MIL) model to act as the affinity model. In [12], the authors proposed using the Support Vector Machine (SVM) to train an affinity model. The SVM performs on a series of low-level features, including the forward-backward optical flow error, the bounding box overlap, the height ratio and the normalized correlation coefficients between the two detections. As these low-level features have less semantic meaning, the SVM is prone to overfitting and needs to be trained for different environments. Recently, deep learning based approaches [13,14,15] have also been used to learn the affinity model. Generally, these models are trained with an end-to-end learning framework and are computationally intensive.

In this paper, we propose a novel compressed deep Convolutional Neural Network (CNN) feature based Correlation Filter (CF) tracker. The CNN feature has abundant geometric and semantic information and is applied in both the object association stage and the lost target re-identification stage. Compared with the point matching based tracking approaches, correlation filter based algorithms have achieved superior performance on several tracking benchmarks [16,17,18]. By combining the CNN feature with the CF tracker, our approach enjoys the merits of both approaches: on the one hand, the tracking module runs faster than 10 Hz due to the efficiency of the CF; and, on the other hand, the compressed CNN feature used in the tracker contains target-specific semantic information. Besides, the feature is directly inherited from the object detection stage, without the need to be re-calculated.

Our main contributions are summarized as follows. Firstly, a multi-scale detector was proposed which can efficiently detect small objects and produces fewer false negatives. Secondly, a novel compressed deep CNN feature based CF tracker, which exploits target-specific semantic information inherited from the detector and has low computational complexity, was. Thirdly, extensive experiments were performed on the KITTI [19] MOT [20] tracking benchmark, and the results show that our approach obtains superior performance to state-of-the-art algorithms.

The rest of the paper is organized as follows: Section 2 introduces the related work. Section 3 describes the details of our approach. Section 4 presents the experimental results and a comparison with state-of-the-art algorithms. Section 5 concludes the paper.

## 2. Related Work

Existing multi-object tracking approaches mostly use a two-step procedure consisting of the detection module and the tracking module. In this section, we point out related methods of these modules.

### 2.1. Object Detection

Recently the performance of object detection approaches has improved substantially. Viola et al. [21] utilized AdaBoost as the detector with Haar-like wavelets features. Dalal et al. [22] proposed linear SVM based approach with feature of Histogram of Oriented Gradient (HOG). To alleviate the non-rigid deformation effect, Felzenszwalb et al. [23] introduced an object detection system based on the latent SVM. Current state-of-the-art object detectors are mostly derived from R-CNN (Regions with CNN features) [1,2]. These approaches integrate the feature extraction and object detection into an end-to-end learning framework. These approaches could be roughly divided into two categories: Region Proposal Networks (RPN) based approaches and Single Shot multibox Detector (SSD) based approaches. RPN based methods are composed of two neural network branches: the region proposal network and the classification network [3,4]. These two networks are usually trained separately. For the SSD approach, it directly encapsulates the proposal generation and the classification into a single deep neural network, thus resulting in a much faster detection speed while simultaneously achieving similar accuracy [6] with RPN based approaches.

However, both approaches encounter difficulties in detecting small objects. For the RPN based method, due to the low resolution of the region proposal layer, it could hardly generate small object proposals. This is known as the collapsing bins problem [5]. For the SSD based approach, it directly generates object proposals from multiple layers, and the small object proposals are usually generated from shallow layers which contain mostly low-level features. Therefore, the generated small object proposals usually contain many false negatives. In this paper, we reduce the false negatives of small objects by incorporating temporal ROIs.

### 2.2. Object Tracking

After the potential objects are detected, they will be linked in nearby frames to form tracklets. This procedure is usually known as the data association. The literature on solving the data association problem is vast, including Linear Program based approaches [24], Bayesian filtering based approaches [25,26], graphical model based approaches [10,27,28,29,30,31], etc. However, most of these approaches are designed for offline usage. To meet the online need for the autonomous vehicle, some approaches [12,32] model the data association problem as a causally optimal assignment problem and solve it with the Hungarian algorithm [33].

For single object tracking, Kalman filter based approaches [34] and Particle filter based approaches [35] are the most widely known. Besides these state estimation based methods, key point matching based approaches [36,37] are widely used for visual object tracking. Recently, Correlation Filter (CF) based methods [38] have been shown to outperform [18] key point matching based approaches which are frequently used in multi-object tracking area [10,12,25]. Since the seminal work in [38], CF has made great progress, and most state-of-the-art CF trackers have begun to use deep CNN features [18]. The usage of deep CNN features can indeed enhance the tracking performance, but it also brings heavily computational load. Furthermore, as the CNN is usually pre-trained from other datasets, it contains little target-specific semantic information [39]. To that end, we propose a novel deep compressed CNN feature based tracking approach in this paper. The CNN features are shared with the object detection module, thus introducing little computational effort. The CNN channels are also further compressed for more efficiency.

## 3. The Proposed Approach

In this section, we describe our approach. The proposed approach includes two key modules, the multi-scale object detection module and the compressed CNN feature based Correlation Filtering module (CCF). These two modules are interleaved. By carefully integrating these two modules, our proposed multi-object tracking framework could efficiently track multiple objects, and it also has the ability of re-identification (ReID) once the tracked object gets lost.

### 3.1. Multi-Scale Object Detection

The proposed multi-scale object detection module is based on the Single-Shot multibox Detection (SSD) [6] algorithm. We briefly describe this algorithm first. Then, to adapt to online video detection, the fine-tuning process and the usage of temporal information are introduced.

SSD directly produces object bounding box proposals on different convolutional layers with multiple scales $s_{n}$ and different aspect ratios $a_{r}=\left\{1,2,3,\frac{1}{2},\frac{1}{3}\right\}$. The width of the bounding box is $w_{n}^{a}=s_{n}\sqrt{a_{r}}$, and the height is $h_{n}^{a}=s_{n}/\sqrt{a_{r}}$, where $s_{n}$ is the scale factor for the *n*-th convolutional layer, and is calculated as:

(1) $$s_{n}=s_{min}+\frac{s_{max}-s_{min}}{m-1}\left(n-1\right),n\in \left[1,m\right],$$

where $s_{min}$ and $s_{max}$ are set to be 0.2 and 0.9, respectively, across all the experiments. To detect larger objects, the width and height of the default box are set to be $\sqrt{s_{n}s_{n+1}}$.

To effectively detect small objects, the data augmentation strategy is usually adopted. In this strategy, the training data are augmented with patches randomly cropped from the input images. The cropped patches are classified into three categories: intact object, part of object or without object. All these cropped patches are then resized to the same size as the input image. In this way, the small object proposals could be generated in deeper layers. It is well known that deeper layers contain more semantic information, which is essential for reducing false detections.

**Figure 2:**
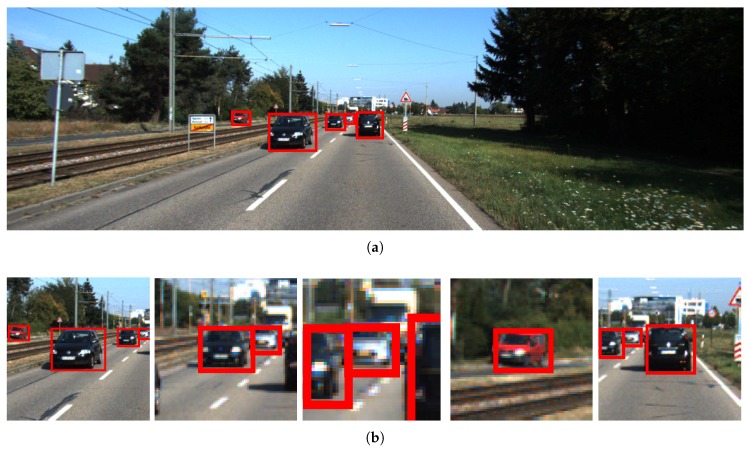
(**a**) The original image with ground-truth bounding boxes overlaid on it; and (**b**) the cropped patches around the object ROIs.

In contrast to augmenting the training data with randomly cropped patches, we augment the training data with object ROI patches. The patches are cropped around objects with size:

(2) $$S=\min\left\{S_{norm}/mean\left(S_{org}\right),2\right\}*mean\left(S_{org}\right),$$

where $S_{org}$ is the object size and $S_{norm}$ is set to be [512,512]. These cropped patches are then fused with the original images to form the augmented training set, as shown in Figure 2. In this way, the SSD training set is augmented with multi-scale samples. We termed this approach as SSD fine-tuned on multi-scale training data.

This data augmentation strategy, however, could only be performed in the training stage. In the detection stage, there is no mechanism to crop object ROIs, and the smaller object proposals could only be generated in the shallow feature layers. When detecting objects in sequential frames, the temporal information can provide an important position prior for detectors. As the object moves smoothly across sequential frames, its position in the previous frame is highly correlated with the position in the current frame. More precisely, we utilize correlation filter to predict object position in the current frame. The details are introduced in the next section. Based on the predicted position, we can crop object ROIs and generate a set of multi-scale input data in the detection stage. As shown in Figure 1, these ROIs are then resized to a fixed size using bilinear interpolation. In this way, the small objects are upsampled and the corresponding proposals could be generated from deeper layers which contain more valuable semantic information. Experiments show that this semantic information helps reduce the false negatives for small object proposals. The details are described in Section 4.2.

### 3.2. Compressed CNN Feature Based Correlation Filter

**Figure 3:**
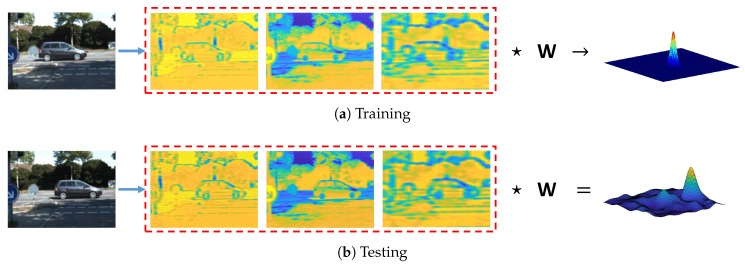
The figure illustrates the training (**a**) and testing (**b**) process of CCF. Three of the compressed CNN feature channels are displayed in the middle. In the training phase, correlation filter weight **W** is obtained by solving the ridge regression problem in Equation (3), and the desired output is a Gaussian distribution. In the testing phase, the response map is calculated using Equation (8).

For the tracking method, we chose to use the correlation filter as our base approach. Let w represent the weights to be learned, which is multiplied by the input sample feature x. The output is the desired filter response y. For a given training image, multiple training samples are generated by circular shift around the target, and the desired output is modeled as a Gaussian distribution with a small variance, as shown in Figure 3. This problem could be cast as a ridge regression problem:

(3) $${min∥w★x-y∥+\lambda ∥w∥}^{2},$$

where ★ represents the circular correlation, and λ is the weight of regularization.

To reduce the computational complexity, the circular correlation in the spatial domain could be transformed into the frequency domain using FFT as:

(4) $$min∥{\hat{w}}^{*}⊙\hat{x}-\hat{y}∥+\lambda ∥\hat{w}{∥}^{2},$$

where $\hat{w}$, $\hat{x}$, and $\hat{y}$ are Fourier transformations of w,x, and y, respectively; and ⊙ denotes element-wise multiplication. ${\hat{w}}^{*}$ is the complex conjugate of $\hat{w}$, which ensures the operator to be correlation instead of convolution [40]. The solution to Equation (4) is:

(5) $$\hat{w}=\frac{{\hat{y}}^{*}⊙\hat{x}}{{\hat{x}}^{*}⊙\hat{x}+\lambda }.$$

To adapt to the object appearance variations, the correlation filter is online updated as:

(6) $$N_{t}=\left(1-\psi \right)A_{t-1}+\psi {\hat{y}}^{*}⊙{\hat{x}}_{t},$$

(7) $$D_{t}=\left(1-\psi \right)D_{t-1}+\psi {\hat{x}}_{t}^{*}⊙{\hat{x}}_{t},$$

where $N_{t}$ and $D_{t}$ are the numerator and denominator of Equation (5), respectively. *t* is the frame index and ψ is the updating rate.

Once the weights $\hat{w}$ are learned in the training stage, they can be used to track the object. The response map for tracking the object is calculated as:

(8) $$y_{o}=F^{-1}\left\{{\hat{w}}^{*}⊙\hat{z}\right\},$$

where $\hat{z}$ is the Fourier transform of the feature input, $F^{-1}$ denotes the inverse Fourier transform, and $y_{o}$ is the response map, as shown in Figure 3.

The computational complexity of the correlation filter is O(NMlogM), where *N* is the number of feature channels and *M* is the length of each feature channel. The original implementation of CF only works with a few channels [38]. These channels correspond to low-level features, including histogram of gradients (HOG), magnitude of gradients, etc. Containing little semantic information, the low-level feature channels hinders the tracking performance of the CF. The ideal feature channels should be compact to ensure computational efficiency and contain discriminative semantic information.

Recently, CNN features have been demonstrated to be discriminative for object tracking [18]. For a typical CNN, it contains both the shallow layers and deep layers. The shallow layers contain geometric information and have high spatial resolution, which is crucial for object localization. A precise object localization is beneficial to the online update of the tracking model. The deep layers contain semantic information that is robust to occlusion, rotation, or illumination variation. The proposed tracker utilizes features from multiple layers of the SSD network, exploiting both geometric and semantic information.

We chose to incorporate the CNN feature into the correlation filter. The CNN layers that we use include *conv1-1*, *conv3-3*, and *conv5-3* from SSD network with 64, 256, and 512 channels, respectively. To efficiently utilize these CNN feature channels, their dimensions need to be compressed. We compressed the feature channels using the Principal Component Analysis (PCA). The coefficient matrix of PCA was calculated once an object was detected and then kept fixed during the whole tracking process. To retain about 90% of variance, the number of the compressed feature channels was set to be 3, 30 and 10, accordingly. In the training stage, three correlation filters were trained with the three compressed feature channels. In the tracking stage, the three trained correlation filters were applied to obtain three response maps, which were combined linearly to obtain the final response map.

By combining the CF with the CNN feature, our proposed tracker enjoys the merits of both approaches. Furthermore, the CNN features we used in the tracker were directly inherited from the object detector, thus required no further computational load.

### 3.3. Multi-Object Tracking with Object Re-Identification

During the tracking step, the targets may disappear for several factors, such as occlusion, moving out of view, etc. When these temporally disappeared objects re-appear, the tracker should have the ability to recognize them, and assign them to existing tracklets instead of initializing a new tracklet. This procedure is usually known as the object re-identification (ReID).

To realize the ability of ReID, we should have a mechanism to judge whether the tracker output is becoming unreliable. Ideally, the response map of the correlation filter should have only one sharp peak and be smooth in other regions. Therefore, the fluctuation degree of the response map is an ideal indicator for the reliability of the tracker. As introduced in [41], we model the degree of fluctuation using the average peak-to-correlation energy (APCE):

(9) $$s_{a}=\frac{y_{o}^{max}-y_{o}^{min}}{mean(\sum_{i=1}^{w\times h}{\left(y_{o}^{i}-y_{o}^{min}\right)}^{2})},$$

where $y_{o}$ is the response map of Equation (8). *w*, *h*, $y_{o}^{max}$, and $y_{o}^{min}$ represent the width, height, and maximum and minimum of $y_{o}$, respectively.

For the data association algorithm, the key points matching [10,12] and the intersection-over-union (IoU) of adjacent detections are usually adopted as the affinity model. We designed an affinity model using both the geometric cue and the appearance cue. The IoU between the position predicted by tracker and the current detection was used as the geometry cue, and the APCE score of the tracker response map was used as the appearance cue. The data association cost matrix is defined as:

(10) $$S_{i,j}=\left\{\begin{array}{c} C\left(t_{i},d_{j}\right),d_{j}>\tau_{1}\\ \infty ,\;otherwise, \end{array}\right.$$

where $C(t_{i},d_{j})$ is the association cost, $t_{i}$ is the *i*-th tracker, and $d_{j}$ is the *j*-th detection. $C(t_{i},d_{j})$ is calculated as:

(11) $$C\left(t_{i},d_{j}\right)=\left(1-IoU\left(t_{i},d_{j}\right)\right)\times \left(1-\mathrm{APCE}\left(t_{i},d_{j}\right)\right).$$

Based on this cost matrix *S*, the data association problem was then modeled as an optimal assignment problem, and solved using the Hungarian algorithm [33].

**Figure 4:**
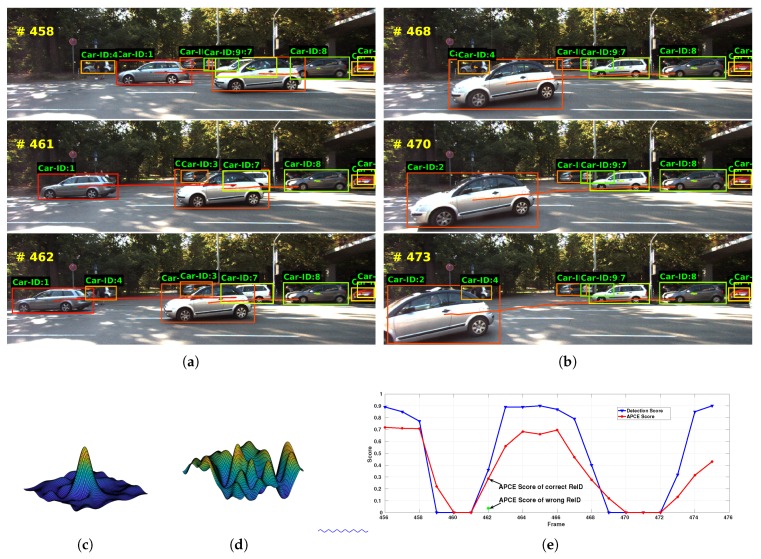
We show the ReID process of our approach using the car of ID-4 as an example. As shown in (**a**), the car ID-4 is firstly detected in frame 458, but then completely occluded in frame 461. After it re-appears again in frame 462, our ReID algorithm correctly recognize it as ID-4. (**b**) also shows an example of the ReID process. Panels (**c**,**d**) are the response maps of the new detection of ID-4 in frame 462 with two inactive tracklets: ID-4 and ID-9. It is observed that (**c**) has a sharp peak and (**d**) fluctuates intensively. By calculating the APCE score of these two score maps, as shown in (**e**), we correctly assign this new detection to ID-4.

We show an illustrative example of our ReID program in Figure 4. In frame 458, two new tracklets, ID-4 and ID-9, are initialized, with their detection scores larger than the threshold $\tau_{1}$. These two tracklets are then completely occluded in frame 461, and their detection scores become zero. Thus, they are labeled as inactive and stored as historical tracklets. In frame 462, vehicle ID-4 re-appears. We calculate the correlative responses of this new detection with existing inactive ID-4 and ID-9 tracklets, and the results are shown in Figure 4c,d. From there, we could observe that, although the maximums of the two responses are similar, their fluctuation degrees are different. The correct ReID (ID-4) response exhibits a single sharp peak, while the wrong ReID (ID-9) response fluctuates intensively. The APCE scores of the two responses in frame 462 are clearly distinguishable, as shown with the red and green point in Figure 4e. This suggests that our ReID mechanism is able to recognize specific target among similar looking objects.

Our proposed framework is summarized as follows. For a new frame, firstly the multi-scale object detection module is used to detect the potential objects. Then, we calculate the correlation coefficients and assign detections to existing tracklets using the Hungarian algorithm. After the assignment, if there are detections left with scores larger than $\tau_{1}$, they are treated as potential new objects. Before assigning new IDs to these objects, the object ReID program is performed. We calculate the APCE scores of the new detections with existing inactive tracklets. If the APCE score $s_{a}$ is larger than $s_{a}^{s}$, we activate the tracklet using this new detection. This suggests that a disappeared target reappears again. Otherwise, we assign a new ID to this object. For the ReID process, $s_{a}^{s}$ is the last saved APCE score of the inactive tracklet. Finally, we update the states of the tracklets using threshold $\tau_{1}$ and $\tau_{2}$. Active tracklets will either be updated if their scores $s_{d}$ are larger than $\tau_{2}$, or become inactive if their scores are smaller than $\tau_{1}$. This whole procedure is also described in Algorithm 1.
**Algorithm 1** Multi-Object Tracking with Compressed CNN Feature Based Correlation Filter**Input:** The sequential frames $I={\left\{I_{i}\right\}}_{i=1}^{N}$. **Output:** The trajectories of targets $O={\left\{O_{i}\right\}}_{i=1}^{M}$.   1:**for** each frame *I* in the sequence **do**  2:  Obtain detections $D^{w}$ in the image *I*;  3:  **for** each tracker *T* in the tracker set $T={\left\{T_{i}\right\}}_{i=1}^{K}$ **do**  4:    Crop ROI patch *R* based on tracking output;  5:    Obtain detections $D^{r}$ in the patch *R*;  6:  **end for**  7:  Do Non-Maximum Suppression (NMS) for $\left\{D^{w},D^{r}\right\}$ and obtain the multi-scale detection results $D={\left\{D_{i}\right\}}_{i=1}^{H}$;  8:  Calculate the correlation coefficients between detections D and trackers T;  9:  Associate D and T using the Hungarian algorithm;10:  **for** each detection *D* in D **do**11:    **if** *D* associate with no existing trackers and the score of the detection $s_{d}>=\tau_{1}$ **then**12:      Generate a potential new tracker $T_{new}$ from *D*13:      **for** each inactive tracker $T^{\prime }$ in the inactive tracker set $T^{\prime }$ **do**14:        Calculate the APCE score $s_{a}$ of $T_{new}$ with $T^{\prime }$;15:        **if** $s_{a}>s_{a}^{s}$ **then**16:          Activate $T^{\prime }$ with $T_{new}$;17:        **else**18:          Assign $T_{new}$ with a new target ID;19:        **end if**20:      **end for**21:    **end if**22:  **end for**23:  **for** each tracker *T* in T **do**24:     **if** the score of tracker $s_{d}<\tau_{1}$ **then**25:       *T* becomes inactive, and save the APCE score $s_{a}$ as $s_{a}^{s}$. $T^{\prime }⇐T$;26:     **end if**27:     **if** the score of tracker $s_{d}>\tau_{2}$ **then**28:       Update the CCF model of *T*;29:     **end if**30:  **end for**31:  Save the trajectories of targets: O=O∪T;32:**end for**

## 4. Experiments

### 4.1. Experimental Setup

We performed experiments on the KITTI and MOT tracking benchmark, and implemented the algorithm on an Intel 3.00 GHz CPU and GeForce GTX 1080Ti GPU with MATLAB.

**Datasets and Evaluation Metrics.** The KITTI tracking dataset contains training and testing set with 21 and 29 sequences, respectively. The MOT tracking dataset contains 11 training sequences and 11 testing sequences. For these datasets, only the ground-truth of the training set is publicly available. The results for the testing set should be submitted for online evaluation [19,20].

We split the KITTI training set into a small training set with 6 sequences and a validation set with 15 sequences. Parameters were trained on the small training set, and then analyzed on the validation set.

The performance evaluation metrics include the Mostly Track targets (MT), Mostly Lost targets (ML), ID F1 score (IDF1), Multiple Object Tracking Accuracy (MOTA), and Multiple Object Tracking Precision (MOTP) [42,43], where MT and ML account for the number of ground-truth trajectories that are covered by the tracking output at a ratio larger than 80% and smaller than 20%, respectively. IDF1 is the ratio of correctly identified detections over the average number of ground-truth and computed detections [20]. MOTA is defined as:

(12) $$MOTA=1-\frac{\sum_{t}^{N}\left(m_{t}+fp_{t}+mm_{t}\right)}{\sum_{t}^{N}g_{t}},$$

where $m_{t}$, $fp_{t}$ and $mm_{t}$ denote the number of missed detections, the false positives and the mis-matched objects in frame *t*, respectively. MOTP is defined as:

(13) $$MOTP=1-\frac{\sum_{t}^{N}\sum_{i}^{M}\left(d_{t}^{i}\right)}{\sum_{t}^{N}c_{t}},$$

where $d_{t}^{i}$ denotes the distance between the *i*-th ground-truth and the corresponding tracked object in frame *t*. $c_{t}$ is the number of matches in frame *t*.

**Figure 5:**
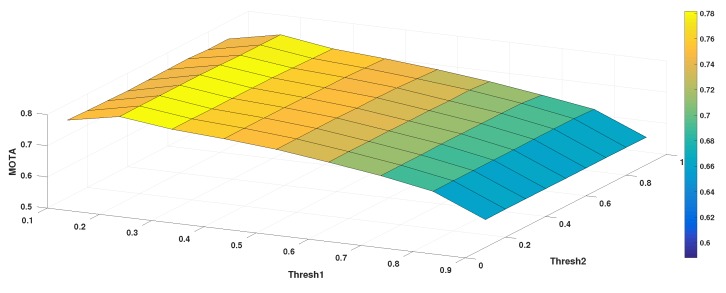
The MOTA value in relation to $\tau_{1}$ and $\tau_{2}$ on the training set.

**Parameters Setting.** In Algorithm 1, the birth/death of a tracker is determined by the threshold $\tau_{1}$. The update frequency of the CCF model is determined by the threshold $\tau_{2}$. These thresholds are achieved by exhaustive search on the small training set, where we used MOTA as the performance indicator. The results are shown in Figure 5. The maximal MOTA was achieved when $\tau_{1}$ was set to 0.2 and $\tau_{2}$ was set to 0.6. The update rate of the CCF model in Equations (6) and (7) was set to 0.0025, as suggested in [44].

### 4.2. Ablation Analysis

**Multi-scale Augmentation.** As shown in Table 1, to demonstrate the effectiveness of the multi-scale augmentation strategy for training data, we compared the performance of various detectors with the same tracker. The detectors included the original SSD, SSD fine-tuned on the training data (Finetuned SSD${}_{O}$), and SSD fine-tuned on the multi-scale augmented training data (Finetuned SSD${}_{OP}$). The training data used were the KITTI detection training set. Compared with the original SSD, Finetuned SSD${}_{O}$ improved the MOTA, MOTP, and MT by 47.25%, 9.47%, and 54.64%, respectively, and decreased ML by 28.45%. This significant improvement clearly suggests that a fine-tuning step is crucial for data-driven approaches. Compared with Finetuned SSD${}_{O}$, Finetuned SSD${}_{OP}$ improved MOTA and MT by 18.09% and 27.06%, respectively, and decreased ML by 14.94%. This performance improvement should be largely attributed to the multi-scale augmentation strategy for the training data.

**Figure 6:**
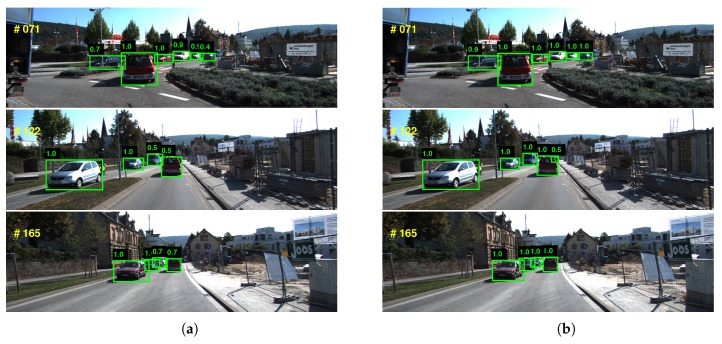
The left column (**a**) shows the results of the fine-tuned SSD, and the right column (**b**) shows the results of the fine-tuned SSD augmented with temporal ROIs. Compared with the fine-tuned SSD, the temporally augmentation based method increases the confidence of positive detections and improves the precision of detection bounding boxes. Besides, the method decreases the false negatives for small objects.

To demonstrate the effectiveness of our temporally augmenting strategy in the detection stage, we evaluated the quantitative performance of various detectors. The results are shown in Table 2 and Figure 6. The original FasterRCNN [3] and fine-tuned SSD detectors were regarded as baseline approaches. The detector named MultiFasterRCNN is the original FasterRCNN augmented with temporal ROIs. The detector named as Finetuned MultiSSD is the fine-tuned SSD augmented with temporal ROIs. Compared with the baseline, the *temporally augmenting methods* obtained better performance. The improvement should be largely attributed to the incorporation of temporal information.

**Affinity Model.**Table 3 compares the performance of the correlation filter based affinity model with the MDP [12] tracker. Compared with MDP, the HOGCF (Correlation Filter with HOG features) based method increased the MOTA by about 11.75%. This suggests that the correlation filter is a better affinity model than the key point matching based approach used in MDP.

We compared the original implementation of correlation filter HOGCF with our compressed CNN feature based CF tracker (CCF). Results in Table 3 show that CCF achieves a 25.42% increase in MT and 4.6% decrease in ML. This suggests that the usage of compressed CNN feature is indeed much better than the low-level HOG features.

### 4.3. Benchmark Evaluation Results

**Results on KITTI Dataset.** The results on KITTI tracking testing set are summarized in Table 4. We compared our algorithm to several state-of-the-art approaches, including SSP [28], DCO-X [27], LP-SSVM [30] and NOMT-HM [10]. Some of these approaches could only be performed in the offline setting.

**Figure 7:**
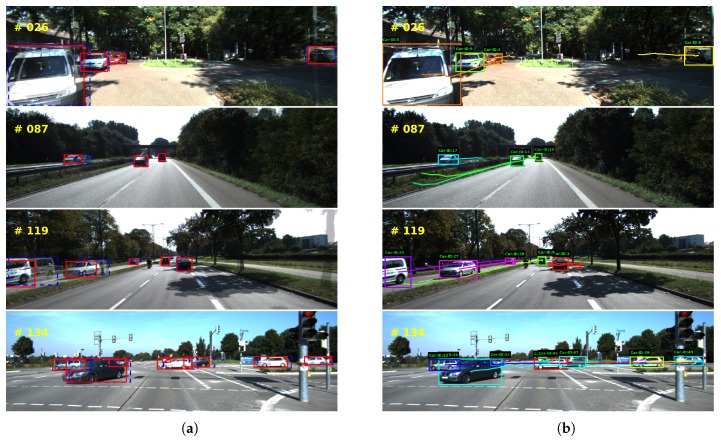
Experimental results on KITTI dataset. Left column (**a**) shows the tracking results with CCF, where we merged two sequential frames to make their difference apparent. The previous position are denoted with blue rectangle and the current with red. Right column (**b**) shows object trajectories. Extensive experiments were performed on dataset with various illuminations, traffic flows, and road conditions, and the results demonstrate our approach is effective for autonomous vehicle to track multi-objects.

For the online setting, our approach achieved the best result. Compared with the second best, we obtained 3.11% and 2.28% increase in MOTA and MOTP, respectively, and 38.0% decrease in ML. The decrease in ML indicates that our approach has fewer false negatives, which should be largely attributed to the proposed temporal object ROIs. Low missing rate is a crucial property for autonomous vehicles to avoid the miss-detection of some important targets, such as pedestrians or vehicles. For the offline setting, our approach outperforms its counterparts in MOTP, MT and ML. Although the offline method LP-SSVM achieves the best result in MOTA, it utilizes the whole sequence for trajectory generation without considering causality, which is impractical for the autonomous vehicle. Figure 7 shows some qualitative results of our approach.

**Results on MOT2015 Dataset.** We compared our algorithm with the state-of-the-art approaches on MOT2015 dataset. The results are summarized in Table 5.

Our approach achieved the best result in MOTA, MT and ML. MDP [12] achieved a better result in IDF1, while it performed much worse in MT and ML. Besides, the MDP tracker needed to be trained for different environments and cannot work in real-time [12]. The oICF tracker [11] is based on Multiple-Instance Learning, and CNNTCM [15] utilizes Siamese CNN to associate objects. The approach of MCFPHD [29] generates the trajectory without considering causality. Compared with these approaches, our approach achieved better performance for MOTA, MT and ML.

**Figure 8:**
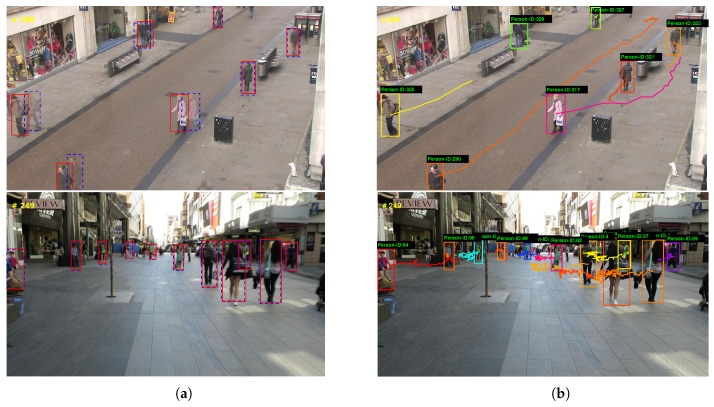
Experimental results on MOT2015 dataset. Left column (**a**) shows the tracking results with CCF, and right column (**b**) shows object trajectories. The experimental results demonstrate our approach is effective for tracking small objects.

MOT2015 dataset contains mostly pedestrian targets which are generally smaller than the vehicle objects in the KITTI dataset. As our approach achieved much better performance in MT and ML, it suggests that our approach is effective in tracking small objects with lower false negatives, as shown in Figure 8.

## 5. Conclusions

In this paper, we propose an efficient algorithm for multi-object tracking. Our approach is a two-step procedure. Firstly, we detect objects using a multi-scale detector. The detection module is augmented with temporal information and is quite effective for detecting small objects. In the second step, we associate the detections across frames. A novel compressed deep CNN feature based correlation filter is proposed as the affinity model. Extensive experiments were performed on the KITTI and MOT tracking benchmarks. Results show that the multi-scale detector helps reduce false negatives. It is also demonstrates that the correlation filter is a more suitable affinity model than previous key points matching based approaches. The compressed CNN feature contains more valuable semantic information than traditional low-level features, such as HOG, and this semantic information is beneficial for increasing the tracking performance.

## Figures and Tables

**Table 1:** Analysis of training data augment strategy on validation set.

Method	MOTA ↑	MOTP ↑	MT ↑	ML ↓
SSD + HOGCF	36.68%	75.90%	21.96%	11.88%
Finetuned SSD${}_{O}$ + HOGCF	54.01%	83.09%	33.96%	8.50%
Finetuned SSD${}_{OP}$ + HOGCF	**63.78%**	**84.77%**	**43.15%**	**7.23%**

**Table 2:** Analysis of temporal ROIs augment strategy on validation set.

Method	MOTA ↑	MOTP ↑	MT ↑	ML ↓
FasterRCNN + HOGCF	35.63%	72.55%	11.37%	21.96%
MultiFasterRCNN + HOGCF	**43.58%**	**77.08%**	**15.50%**	**20.67%**
Finetuned SSD + HOGCF	63.78%	84.77%	43.15%	7.23%
Finetuned MultiSSD + HOGCF	**69.71%**	**85.10%**	**47.80%**	**6.71%**

**Table 3:** Tracking results on the validation set with various trackers.

Method	MOTA ↑	MOTP ↑	MT ↑	ML ↓
Finetuned MultiSSD + MDP [12]	62.38%	**85.64%**	28.16%	21.19%
Finetuned MultiSSD + HOGCF [44]	69.71%	85.10%	47.80%	6.71%
Finetuned MultiSSD + CCF	**71.59%**	84.54%	**59.95%**	**6.40%**

**Table 4:** Comparison with state-of-the-art methods on the testing subset of KITTI dataset.

Method	Sensor	Causality	MOTA ↑	MOTP ↑	MT ↑	ML ↓	Tracking Time(s) ↓
SSP [28]	monocular	online	67.00%	79.00%	41.00%	9.00%	0.60
DCO-X [27]	monocular	offline	68.11%	78.85%	37.54%	14.15%	0.90
NOMT-HM [10]	monocular	online	69.12%	80.10%	38.54%	15.02%	0.09
LP-SSVM [30]	monocular	offline	**77.20%**	77.80%	43.10%	9.00%	0.05
Ours	monocular	online	71.27%	**81.83%**	**48.31%**	**5.85%**	0.07

**Table 5:** Comparison with state-of-the-art methods on the testing subset of MOT2015 dataset.

Method	Sensor	Causality	MOTA ↑	IDF1 ↑	MT ↑	ML ↓	Tracking Time(s) ↓
MDP [12]	monocular	online	30.3%	**44.7**%	13.0%	38.4%	0.91
MCFPHD [29]	monocular	offline	29.9%	38.2%	11.9%	44.0%	0.08
CNNTCM [15]	monocular	offline	29.6%	36.8 %	11.2%	44.0%	0.59
oICF [11]	monocular	online	27.1 %	40.5%	6.4%	48.7%	0.71
Ours	monocular	online	**32.7**%	38.9%	**26.2%**	**19.6%**	0.09
